# Glycosylation of *Trypanosoma cruzi* TcI antigen reveals recognition by chagasic sera

**DOI:** 10.1038/s41598-020-73390-9

**Published:** 2020-10-02

**Authors:** Niamh Murphy, Barrie Rooney, Tapan Bhattacharyya, Omar Triana-Chavez, Anja Krueger, Stuart M. Haslam, Victoria O’Rourke, Magdalena Pańczuk, Jemima Tsang, Jack Bickford-Smith, Robert H. Gilman, Kevin Tetteh, Chris Drakeley, C. Mark Smales, Michael A. Miles

**Affiliations:** 1 Faculty of Infectious and Tropical Diseases, London School of Hygiene and Tropical Medicine, London, UK; 2 Centre for Molecular Processing, School of Biosciences, University of Kent, Canterbury, Kent UK; 3 TroZonX17, Kent, UK; 4 Instituto de Biología, Universidad de Antioquia, Medellín, Colombia; 5 Department of Life Sciences, Imperial College London, London, SW7 2AZ UK; 6 Department of International Health, Johns Hopkins Bloomberg School of Public Health, Baltimore, USA

**Keywords:** ELISA, Diagnostic markers, Parasitic infection

## Abstract

Chagas disease is considered the most important parasitic disease in Latin America. The protozoan agent, *Trypanosoma cruzi*, comprises six genetic lineages, TcI-TcVI. Genotyping to link lineage(s) to severity of cardiomyopathy and gastrointestinal pathology is impeded by the sequestration and replication of *T. cruzi* in host tissues. We describe serology specific for TcI, the predominant lineage north of the Amazon, based on expression of recombinant trypomastigote small surface antigen (gTSSA-I) in the eukaryote *Leishmania tarentolae*, to allow realistic glycosylation and structure of the antigen. Sera from TcI-endemic regions recognised gTSSA-I (74/146; 50.7%), with no cross reaction with common components of gTSSA-II/V/VI recombinant antigen. Antigenicity was abolished by chemical (periodate) oxidation of gTSSA-I glycosylation but retained after heat-denaturation of conformation. Conversely, non-specific recognition of gTSSA-I by non-endemic malaria sera was abolished by heat-denaturation. TcI-specific serology facilitates investigation between lineage and diverse clinical presentations. Glycosylation cannot be ignored in the search for immunogenic antigens.

## Introduction

American trypanosomiasis (Chagas disease) is a neglected tropical disease caused by the protozoan parasite *Trypanosoma cruzi*, and is considered the most important human parasitic infection in Latin America. The infection is transmitted by blood sucking triatomine bugs through contamination of human mucous membranes, abraded skin or food, with *T. cruzi* infected triatomine faeces. Once acquired, unless successfully treated, *T. cruzi* usually persists as a life-long infection, which can also be transmitted congenitally from mother-to-child, and by transfusion or organ donation. Principal human tissues damaged are the heart and the intestinal tract, and symptoms may develop years after the early infection. An estimated 30% of *T. cruzi* infected individuals develop chagasic cardiomyopathy and a proportion of those develop chagasic gastrointestinal megasyndromes^1^. Only two drugs are currently available for treatment, benznidazole and nifurtimox. Due to severe dermatological side effects, treatment of adults frequently fails; a shorter, lower dose schedule for benznidazole and a paediatric formulation have recently been introduced^2,3^.

Currently WHO estimates 5–6 million cases of *T. cruzi* infection worldwide^4^. Despite international control programmes, there is still a high prevalence and incidence in regions such as the Gran Chaco of Bolivia and Argentina. Triatomine vectors are spreading into periurban sites, and Chagas disease is becoming a global health issue among Latin American migrant populations, with an estimated 250,000 infected in the USA, more than 100,000 in Europe and 12,000 in the UK, with risk of global non-vector borne transmission, congenitally and via blood and organ donors^5,6^.

*Trypanosoma cruzi* comprises six genetic lineages TcI-TcVI^7–9^, with TcBat proposed as a seventh lineage related to TcI^10^. Based on genotyping, TcI is the predominant agent of Chagas disease north of the Amazon, with TcIV a secondary cause in Venezuela^11^. TcII, TcV and TcVI are prevalent among cases in the Southern Cone countries of South America (Argentina, Bolivia, Brazil, Chile, Paraguay and Uruguay); TcIII is uncommonly found in human infections^1^.

In 1981^12^ it was proposed that the different geographical distributions of the *T. cruzi* lineages may contribute to the disparate clinical presentations of Chagas disease in the Southern Cone countries, where megasyndromes are found, compared to northern South America, where they are not reported^1^. However, it is complex to prove such an association by parasite genotyping, because *T. cruzi* blood parasitaemia is scanty in chronic Chagas disease, does not necessarily represent lineages sequestered in the internal organs^13–16^, and growth rate competition occurs between isolates grown in vitro.

One approach to surveillance of clinical, geographical and ecological distributions of the *T. cruzi* lineages is to develop lineage-specific serology, originally proposed by Di Noia et al.^17^. Specific epitopes of the *T*. *cruzi* trypomastigote small surface antigen (TSSA), a cell surface mucin, have been identified for all six genetic lineages, with the hybrid lineages TcV and TcVI having two epitopes encoded at the heterozygous locus, one of which is shared with TcII, as shown by Bhattacharyya et al.^18^. Lineage-specific serology with synthetic peptides representing the TcII/V/VI and TcV/VI epitopes enabled surveillance of chagasic patients^19^, and the discovery of reservoir hosts^20,21^. Furthermore, TcII/V/VI serology, adaptable to rapid diagnostic test (RDT) format, demonstrated that among Bolivian patients stratified by severity of cardiomyopathy, TcII/V/VI seropositives were five-fold more prevalent in the severe versus no evidence of cardiomyopathy groups^22^. RDTs also identified TcII/V/VI seropositive sympatric humans and dogs in the Argentine Chaco^23^.

A long-standing research objective is the validation of a robust and sensitive TcI-specific antigen that would enable the enigma of link between infective lineage and clinical prognosis to be more comprehensively investigated. Furthermore, this would enable systematic low-cost analysis of *T. cruzi* transmission cycles and evaluation of the risk of emergence of sylvatic lineages into the domestic environment. However, repeated attempts have failed to develop a lineage-specific serological test for the TcI specific epitope, either using an *E. coli*-expressed recombinant protein or a synthetic peptide^19,24–26^.

Here, we have expressed the TSSA-I epitope within a related trypanosomatid, *Leishmania tarentolae,* which enables O-linked and N-linked glycosylation^27,28^ to determine whether glycosylation and/or structural integrity impart serological recognition of this TcI antigen.

## Methods

### Ethics

All human sera used here were archived, with consent for research, were anonymised, coded, and did not reveal patient identities. Informed consent was obtained from all subjects or, if subjects are under 18, consent was provided by a parent and/or legal guardian. No samples were collected specifically for this work.

Colombian (Bogotá), Venezuelan and Ecuadorean samples: these were collected as part of routine diagnostic examination, with local institutional ethical approvals Universidad de los Andes, Bogotá, Colombia; (Instituto de Medicina Tropical, Caracas, Venezuela; Pontificia Universidad Católica del Ecuador, Quito, Ecuador) and in accord with EC ethical standards, established as part of the ChagasEpiNet international collaboration (ethical approval from London School of Hygiene and Tropical Medicine, UK). Colombian (Medellín) ethical approval was provided by the Instituto de Biología, Universidad de Antioquia, Medellín, Colombia.

Peruvian and Bolivian samples: the following institutional review boards granted ethical approval: Johns Hopkins Bloomberg School of Public Health, USA; Hospital Universitario Japonés, Bolivia; Universidad Católica Boliviana, Bolivia; Universidad Peruana Cayetano Heredia, Peru; Asociación Benéfica Proyectos en Informatica, Salud, Medicina y Agricultura, Peru; and the Centers for Disease Control and Prevention, USA.

Gambian malaria sera were provided, with consent for further research on diagnostics, from London School of Hygiene and Tropical Medicine archives.

Non-endemic control sera were provided at the London School of Hygiene and Tropical Medicine and used with consent for further research on diagnostics.

### Sources of chagasic sera

Chagasic sera were generously provided from the following sources: Colombia (Medellín, n = 55; Bogotá, n = 31); Ecuador (n = 14); Venezuela (n = 4), Peru (n = 42) and Bolivia (n = 10).

### Assessing serological recognition of synthetic peptides by ELISA

Colombian (Medellín) sera were assayed by ELISA with synthetic peptides TSSApep-I, -II/V/VI, -III, -IV, -V/VI, according to protocols described previously^19^. Colombian (Bogotá), Ecuadorean and Venezuelan sera were previously assayed with synthetic peptides TSSApep-I, -II/V/VI, -III, -IV, -V/VI by ELISA; all were negative with TSSApep-I^19^; Peruvian and Bolivian sera were previously assayed by TSSApep-II/V/VI RDT^﻿22^.

### Prediction of glycosylation sites within gTSSA-I recombinant

In order to determine the presence of O and N glycosylation sites on the TSSA-I epitope, the amino acid sequence was submitted to NetOGlyc 4.0 (www.cbs.dtu.dk/services/NetOGlyc/) and NetNGlyc 1.0 (www.cbs.dtu.dk/services/NetNGlyc/) online servers. Prediction included coverage of the SUMO component of gTSSA-I.

### *L. tarentolae* production of recombinant antigens gTSSA-I and gTSSA-II/V/VI

**Figure 1:**
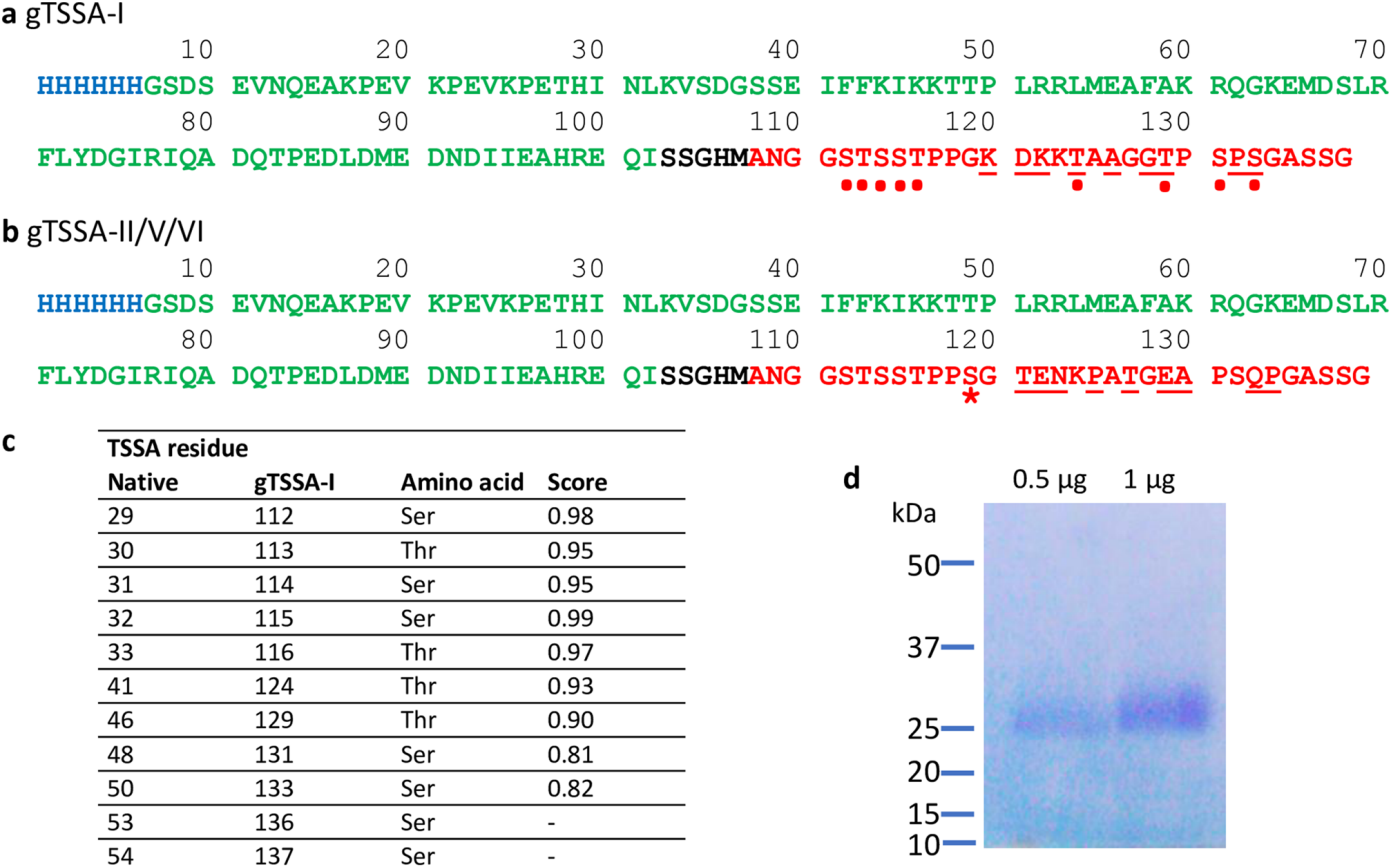
*T. cruzi* TSSA recombinant proteins produced in *L. tarentolae* expression system. (**a**) gTSSA-I protein sequence (based on GenBank GU059925) and (**b**) gTSSA-II/V/VI protein sequence (based on GenBank GU075675): histidine tag (blue), SUMO sequence (green), linker sequences (black) and TSSA sequences (red). Dots: Residues predicted to be O-glycosylated. Starred: Ser residue present in native TSSA-II/V/VI but absent in native TSSA-I. Underlined: lineage-specific polymorphic residues. (**c**) Predicted likelihood scores for O-glycosylation of TSSA-I sequence range between 0 (least)—1 (highest); scores below 0.5 not listed. (**d**) Coomassie blue stained gel of purified gTSSA-I.

The TSSA-specific sequences were cloned into separate expression plasmids pLEXSY_I-blecherry3 with an upstream His tag and SUMO fusion partner to aid solubility of the resulting recombinant proteins, hereafter called gTSSA-I or gTSSA-II/V/VI, expressed in the *L. tarentolae* system (Jena Biosciences, Germany). Figure 1a,b depict the sequences of these recombinant antigens, with N-terminal histidine tag (blue), SUMO sequence (green) and TSSA-I sequence (red).

Production of recombinant protein, based on the methodology of Rooney et al.^29^ was carried out in 1 L baffled Erlenmeyer flasks in BHI medium (supplemented with antibiotics and hemin) and the medium was harvested when the OD_600_ reached 4 (approx. 70 h post inoculation, 10^8^ cells/ml). All media components were from Jena Bioscience. Clarified medium was concentrated 20- fold on a Pellicon XL 50 Ultrafiltration cassette (10 kDa MWCO) and diluted four times in binding buffer (20 mM Phosphate, 500 mM NaCl, 10 mM Imidazole) before addition to an equilibrated His Trap (GE Healthcare) FPLC column. Bound proteins were eluted using an increasing Imidazole gradient (20 mM Phosphate, 500 mM NaCl, 500 mM Imidazole). Peak protein containing fractions, as determined by A_280_ nm measurement, were combined, desalted and concentrated by centrifugation in Amicon Ultra-15 device (5 kDa MWCO). Purified recombinant gTSSA-I was run on Coomassie blue-stained pre-cast 4–12% BisTris gradient SDS-PAGE gel (Novex) using the MOPS running system, along with Precision Plus Protein Unstained Standards (BioRad). The final protein was stored at 1 mg/ml in solution in PBS, 15% glycerol at – 20 °C.

### Glycoproteomics

For glycoproteomics 20 µg of the glycoprotein with the gTSSA-I epitope were denaturated in 10 µL 8 M guanidine hydrochloride solution and reduced with 1 µL of 10 mM DTT (Thermo Fisher Scientific) in 50 mM ammonium bicarbonate buffer (pH 8.4) for 30 min at 56 °C. 1 µL of 55 mM iodoacetamide (Thermo Fisher Scientific) was added and incubated at room temperature for 30 min in the dark. Finally, the digest was diluted with 30 µl of 50 mM ammonium bicarbonate buffer prior to adding 1 µL of trypsin (1:20 w/w enzyme:protein ratio).

The online ESI-LC–MS data were recorded in MSE mode for 60 min using a Waters Synapt G2 mass spectrometer (Waters, Milford, MA). Separations were achieved on C18 Acquity UPLC M-Class column (HSS T3 1.8 µm 75 µm × 150 mm) equilibrated at 40 °C. The mobile phases were A: 0.1% (v/v) formic acid (Biosolve Chemicals) in LC–MS grade water (Greyhound) and B: 0.1% (v/v) formic acid (Biosolve Chemicals) in acetonitrile (Greyhound). Lockmass was set to m/z: 785.80 Glu-1-Fibrinopeptide B (200 nmol/µl, Waters). The Synapt G2-S mass range was operated between 50–2000 m/z.

Initial peptide mapping was carried out using BiopharmaLynx 1.3.3 (Waters, Milford, MA) to locate the epitope region of the gTSSA-I construct in the chromatogram. Interpretations of glycopeptide MS data were performed manually by previously reported methods based on amino acid and sugar masses together with the known fragmentation of glycopeptides^30^, with the assistance of MassLynxV4.1. (Waters, Milford, MA).

### Assessing serological efficacy of the recombinant antigen gTSSA-I

Separate wells of a 96-well flat bottomed ELISA plates (735–0465: Immulon 4HBX, VWR) were coated with 50 μl/well of 1 µg/ml *L. tarentolae*-expressed gTSSA-I, and with 100 μl/well of 2 µg/ml lysate of *T. cruzi* TcII strains IINF/PY/00/Chaco23 or MHOM/BR/00/Y in coating buffer (15 mM Na_2_CO_3_, 34 mM NaHCO_3_, pH 9.6). Lysates were prepared as described previousy^19^. After overnight incubation at 4 °C, plates were washed three times with PBS/0.05% (v/v) Tween 20 (P7949: Sigma Aldrich, UK) (PBST), then blocked with 200 μl of PBS/2% skimmed milk powder (PBSM) at 37 °C for 2 h. Following three washes, 100 μl of 1:200 dilutions of serum in PBSM with 0.05% Tween 20 (PBSMT) was applied and incubated at 37 °C for 1 h; after six further washes, 100 μl of donkey anti-human IgG (H + L)-HRP (709–035–149: Jackson ImmunoResearch, USA) diluted 1:2000 (Peruvian and Bogotá sera) or 1:4000 (Medellín, Ecuadorean, Venezuelan, Bolivian sera) in PBSMT was added and incubated at 37 °C for 1 h. Following six washes, wells were developed with 100 µl/well of 50 mM phosphate/citrate buffer (pH 5.0) containing 2 mM o-phenylenediamine HCl (P1526: Sigma Aldrich) and 0.005% (vol/vol) H_2_O_2_ (216763: Sigma Aldrich); the plates were incubated in the dark at room temperature for approximately 10 min. Reactions were stopped by 50 µl/well of 2 M H_2_SO_4_, and absorbance values read at a wavelength of 490 nm. For Gambian malaria samples, sera were used at 1:100 and anti-human IgG 1:2000.

### Excluding cross reaction with the SUMO component of recombinant antigens

A subset of 34 Colombian (Medellín) samples were assayed for serological cross reactivity with the SUMO component of the recombinant antigens. This was done by parallel performance of ELISAs, as described above, with the recombinant antigen gTSSA-II/V/VI coated in separate wells from the gTSSA-I.

### Assessing the contribution of glycosylation and secondary structure to antigenicity of gTSSA-I

Contribution of glycosylation to antigenicity was assessed using an assay based on the protocol of Woodward et al.^31^, and employed subsequently^32,33^, which described the oxidative cleavage of carbohydrate vicinal –OH groups and subsequent reduction of generated aldehyde groups to prevent non-specific antibody binding. After the blocking and washing steps of the ELISA, described above, all wells were rinsed in periodate buffer (50 mM sodium acetate buffer, pH 4.5), and the wells that had been coated with gTSSA-I received 5 mM freshly-made sodium (meta)periodate (71859: Sigma Aldrich) in periodate buffer; the remaining wells received periodate buffer only. Plates were incubated in the dark at room temperature for 1 h, followed by rinsing of all wells with periodate buffer. The periodate-treated wells were then reduced with freshly-made 50 mM sodium borohydride (71320: Sigma Aldrich) in PBS for 30 min; the remaining wells received PBS only. Following this step, wells were washed three times with PBST before addition of sera and subsequent processing, as described above.

To investigate the contribution of secondary structure to the antigenicity of gTSSA-I, an aliquot of gTSSA-I in coating buffer was heated > 95 °C for up to 10 min, prior to coating onto the plate and performance of the serological efficacy was assessed as described above.

### Statistical analysis

Replica ELISA plates were run in duplicate simultaneously. Cut-off values were determined by first subtracting the background absorbance values (i.e., mean of wells with coating buffer only, no antigen) from the mean reading for each sample; samples that were then greater than three standard deviations above the mean of seronegative non-endemic controls were considered positive. *P* values were determined by performing two-sample T test either unpaired (gTSSA-I against gTSSA-II/V/VI) or paired (unmodified gTSSA-I against oxidised or denatured) with GraphPad Prism (GraphPad Software, San Diego, USA).

## Results

### Synthetic peptides were not recognised by TcI endemic chagasic sera

All Colombian (Medellín and Bogotá), Ecuadorean, Venezuelan, and northern Peruvian sera samples (n = 146) were from regions considered to be endemic for TcI principally^11^, and were predominantly from chronic cases of Chagas disease in rural locations^1,34^. All sera were seropositive with *T. cruzi* lysate; (TcII and TcI lysate antigens do not discriminative between lineage infections). Apart from Colombia (Medellín) and Peruvian samples, all had been previously assayed with synthetic peptide TSSApep-I, and no reaction had been identified^19^. Here, there was no TSSApep-I recognition by the Colombian sera (Medellín) and no indication of the presence of infection with any of the other synthetic peptides.

### Glycosylation prediction

Figure 1c shows the bioinformatic analysis predicting the likelihood score for O-linked glycosylation of the TSSA-I sequence used in gTSSA-I. No N-linked glycosylation was predicted by NetNGlyc 1.0, nor was any glycosylation predicted on the SUMO component of the recombinant proteins.

### Recombinant antigens produced in *L*. *tarentolae*: gTSSA-I and gTSSA-II/V/VI

Recombinant proteins were produced by the *L. tarentolae* LEXSY system and purified by NiNTA from the culture media. The predicted mass is 15 kDa but a broad smear between 25 and 35 kDa is typical for a glycosylated protein (Fig. 1d, and supplementary Figure S1).

### Mass spectrometry analysis proves glycosylation of recombinant gTSSA-I

Confirmation of glycosylation was demonstrated by mass spectrometry based glycoproteomics. An O-glycosylated glycopeptide was identified, consisting of the sequence TAAGGTPSPSGASSG substituted with a single N-acetylhexosamine (HexNAc) residue (Supplementary Figure S2). Such O-glycosylation with a single HexNAc residue is consistent with previous reports of *L. tarentolae* recombinant glycoprotein glycosylation, confirming the prediction of the NetOGlyc 4.0 programme^28^. As predicted by NetNGlyc 1.0, no N-linked glycosylation was found.

### Recognition of gTSSA-I

Overall (74/146; 50.7%) of chagasic sera from TcI endemic regions of northern South America were seropositive with gTSSA-I (Table 1).

**Table 1:** Recognition of gTSSA-I by chagasic sera from northern South America.

Source	gTSSA-I reactive (%)
Colombia (Medellín)	47/55 (85.5%)
Colombia (Bogotá)	7/31 (22%)
Ecuador	6/14 (42.9%)
Venezuela	2/4 (50%)
Peru	12/42 (28.6%)

### Absence of SUMO cross-reactivity between gTSSA-I and gTSSA-II/V/VI

**Figure 2:**
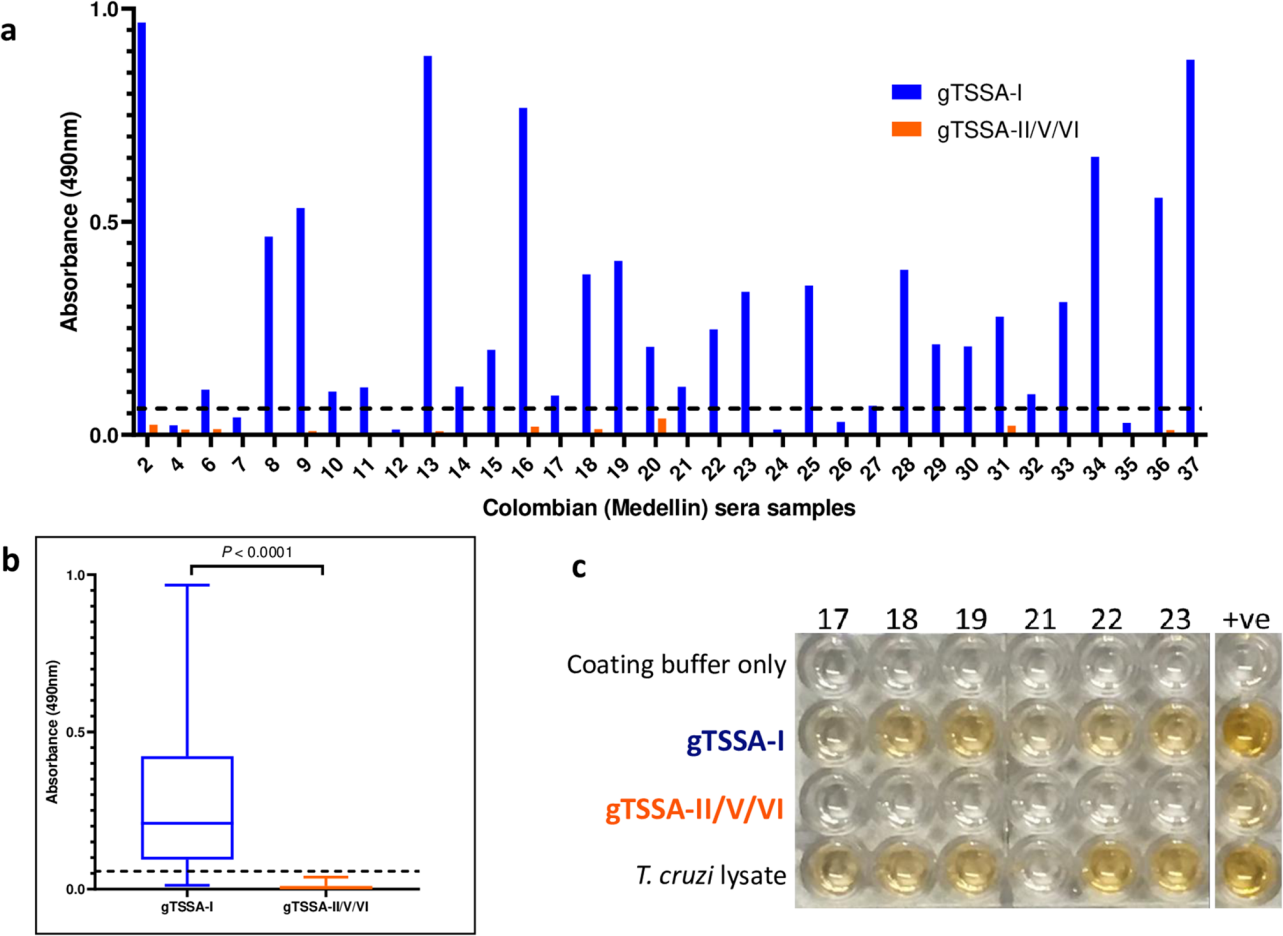
Antigenicity of gTSSA-I is due to TSSA-I sequence. (**a**) Absorbance values with Colombian (Medellín) sera recognising gTSSA-I (blue bars) and gTSSA-II/V/VI (orange bars). (**b**) Overall absorbance values for Colombian (Medellín) sera against gTSSA-I (blue) and gTSSA-II/V/VI (orange); gTSSA-I is recognised, whereas gTSSA-II/V/VI is not (*P* < 0.0001). (**c**) ELISA plate illustrating the recognition of gTSSA-I but not of gTSSA-II/V/VI; all samples were seropositive with lysate, and coating buffer controls were negative. Sample numbers correspond with (**a**). Positive control: serum from a Bolivian patient previously shown to be reactive with synthetic peptide TSSApep-II/V/VI and also seropositive with gTSSA-I, indicating co-infection (see text).

A subset of Colombian (Medellín) sera positive for gTSSA-I (n = 34) were assayed by ELISA against gTSSA-II/V/VI, to assess levels of potential cross-reactivity to the SUMO component of gTSSA-I. No recognition of gTSSA-II/V/VI was observed with these sera (Fig. 2a). Grouping these data, there was a significant difference in the absorbance values between these two recombinant antigens (*P* < 0.0001) (Fig. 2b). Thus, there was no cross reactivity between these two recombinant antigens attributable to antibody recognition of the SUMO component. Bolivian sera shown previously to be reactive with synthetic peptide TSSApep-II/V/VI (n = 5) reacted with gTSSA-II/V/VI; two sera also recognising gTSSA-I (Fig. 2c), suggesting TcII/V/VI and TcI co-infection.

### gTSSA-I antigenicity is principally dependent on glycosylation, not structure

**Figure 3:**
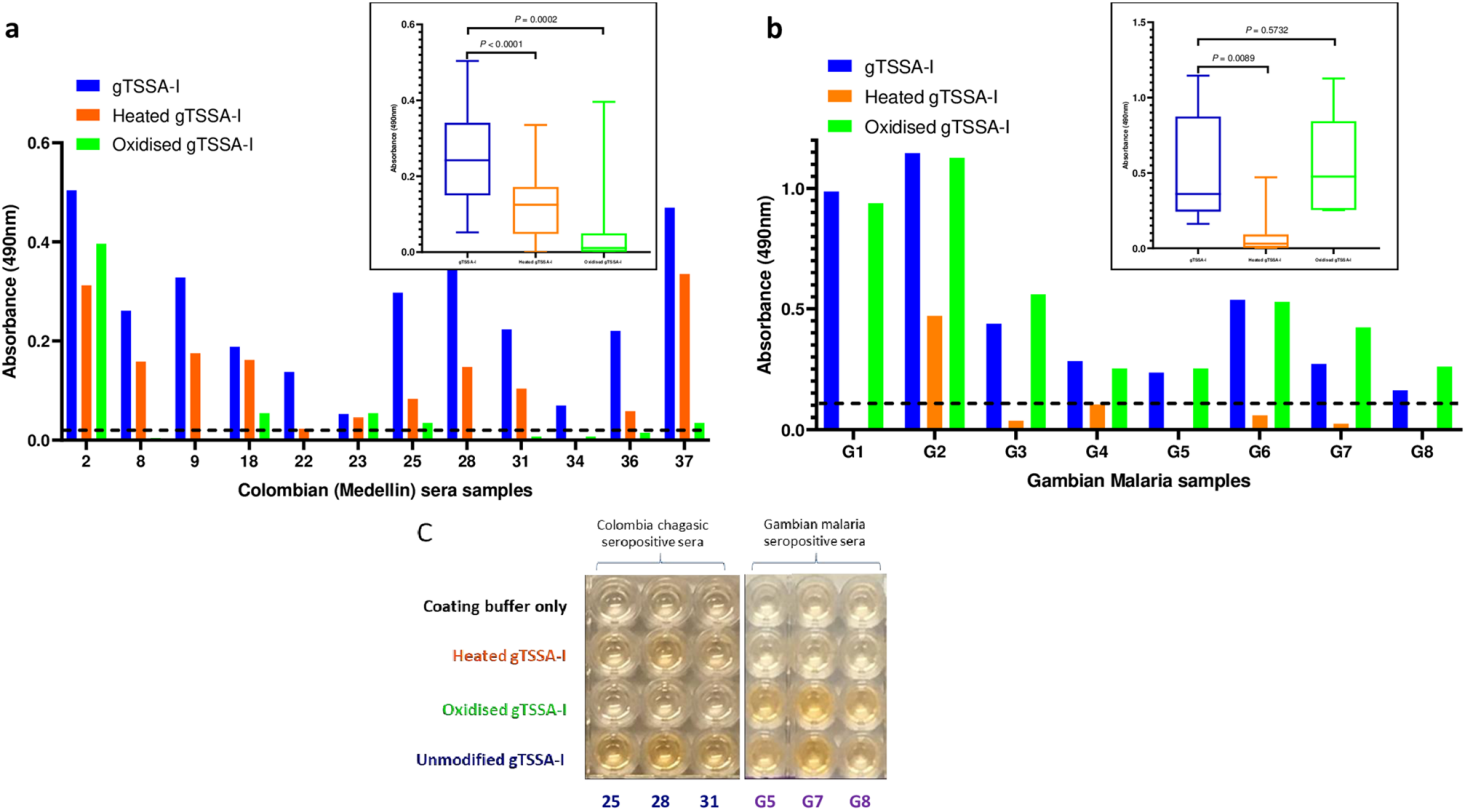
gTSSA-I antigenicity is dependent on glycosylation, whereas recognition by non-endemic malaria sera is dependent on structure. (**a**) Absorbance values of Colombian Medellín sera recognising unmodified gTSSA-I (blue bars), heat denatured gTSSA-I (orange bars) and oxidised gTSSA-I (green bars); oxidation decreases absorbance values substantially more than heat denaturing for all samples except 2. (**b**) Absorbance values of Gambian malaria (G) sera recognising unmodified gTSSA-I (blue bars), heat denatured gTSSA-I (orange bars) and oxidised gTSSA-I (green bars); heat denaturing decreases absorbance values substantially, whereas oxidation does not. For each of the data sets represented in (**a**) and (**b**) individual samples are presented on the main graph and composite absorbance values in the inset box-and-whisker plots. (**c**) ELISA plate illustrating the effects of modifications: coating with of unmodified gTSSA-I (fourth row); heat denatured gTSSA-I (second row), with slight signal reduction for Colombian sera but ablation for Gambian malaria sera; oxidised gTSSA-I (third row), with considerable reduction for Colombian sera, yet no reduction with Gambian malaria sera; coating buffer only (no antigen) control (first row). Sample numbers correspond with (**a**) and (**b**).

A subset of Colombian (Medellín) samples that recognised gTSSA-I (n = 12) were assayed against periodate-treated, heat denatured and unmodified gTSSA-I on the same ELISA plates. Recognition of gTSSA-I decreased substantially after periodate treatment, compared to the heat denatured antigen, for all samples except sample 2 (Fig. 3a). The absorbance values were significantly lower (*P* = 0.0002) after periodate treatment, with only a single sample retaining the same level of recognition as with the unmodified antigen. After heat denaturation the absorbance values of all 12 samples were reduced to a much lesser extent, albeit with a significant difference (*P* < 0.0001) (Fig. 3a).

### gTSSA-I recognition by some Gambian malaria sera is dependent on structure

Unexpectedly, recognition of gTSSA-I was observed with 13/24 Gambian malaria sera samples. Eight of these reactive samples were assayed against periodate-treated (oxidised) and heat denatured gTSSA-I. Oxidation had little effect on recognition of gTSSA-I, with no significant difference between absorbance values (*P* = 0.5732) and recognition of gTSSA-I was retained by all 8 samples. However, this was abolished by heat denaturation of this antigen (*P* = 0.0089). One sample remained above the cut-off, though the absorbance value had been greatly reduced (Fig. 3b,c). Any recognition of gTSSA-I by non-endemic healthy controls (NEHC) was also abrogated by heat-denaturation.

## Discussion

Lineage-specific serology for *T. cruzi* infections provides a powerful tool for understanding the epidemiology and ecology of Chagas disease. Here, we have investigated lineage-specific epitopes to determine the importance of glycosylation in antigen recognition, particularly in relation to TcI.

The *L. tarentolae* expression system has been used previously to produce *Trypanosoma brucei gambiense* surface antigens, as a low cost alternative to harvesting diagnostic antigens from *T. b. gambiense* grown *in vitro*^29^. Expression in *L. tarentolae* has also been applied in attempts to improve diagnostic antigens for the trypanosomatids *Leishmania braziliensis*^35^ and *Leishmania donovani*^36^.

Here, we applied *L. tarentolae* expression to produce recombinant *T. cruzi* gTSSA-I, and to determine whether consequent glycosylation or more *bona fide* structural conformation of the protein conferred serological recognition upon the antigen.

All Colombian, Venezuelan, and Ecuadorean sera that were positive here with gTSSA-I were previously seronegative with the synthetic peptide TSSApep-I. The Medellín samples showed by far the highest proportion that recognised gTSSA-I; 85.5%. Reasons for this are unclear, but may be related to lower levels of anti-*T. cruzi* IgG in the other sera; in some localities there are low IgG antibody levels in *T. cruzi* infections^37^. Furthermore, the gTSSA-I positive samples were not seropositive with gTSSA-II/V/VI, demonstrating that there was no serological cross reaction with the SUMO component of the recombinant antigens. Thus, we have demonstrated clear, robust TcI lineage-specific serology with sera originating from countries where TcI has been identified by genotyping as the principal cause of Chagas disease.

In comparison with synthetic peptides or production of recombinants in the bacterium *E. coli,* heterologous expression in the eukaryote *L. tarentolae* enables glycosylation of the trypanosomatid proteins and adoption of a more natural conformation. O- and N-linked glycosylation of recombinant proteins in *L. tarentolae* has been described, and the wild type pattern in *T. cruzi* also shown to be O-linked (GlcNAc)^28,38^. Assays with periodate-treated antigen showed that the protein glycosylation (predicted by the online algorithms and demonstrated by glycoproteomics), and not the protein conformation, was important for serological recognition of gTSSA-I. Thus, expression in *L. tarentolae* revealed antigenic properties of TSSA-I that are not evident in synthetic peptides or *E. coli*-expressed recombinant proteins.

The specificity of gTSSA-I was questioned, due to some cross-reactivity with non-endemic (Gambian) malaria control sera. The sera from malaria were included due to breadth of immune response associated with antigenic variation, and recent research interest in whether antibodies to *Plasmodium* may recognise glycosylated antigens^39^. Two of these controls were also seropositive with the lateral flow diagnostic test specifically to detect exposure to *T. b. gambiense* infection (data not shown), suggesting an explanation for their cross reactivity. However, heating gTSSA-I abolished recognition by all Gambian malaria sera, demonstrating that this was due to analogous protein structure of gTSSA-I, not glycosylation. Since this is contrary to gTSSA-I recognition by chagasic sera, heating of gTSSA-I prior to performance of diagnostic ELISA or to incorporation into RDTs provides TcI-specific diagnosis via recognition of glycosylation antigenicity.

We have previously demonstrated a link between serological recognition of TSSApep-II/V/VI and severity of chagasic cardiac symptoms^19,22^. Availability of gTSSA-I serology enables parallel investigation of clinical status associated with TcI infection, and serological detection of sporadic TcII/V/VI and TcI co-infections, which occur in some Bolivian and Brazilian endemic foci^40,41^. As with application of TSSA-II/V/VI serology to sylvatic mammals^20,21,23,42,43^, gTSSA-I can be deployed for resolution of TcI domestic and sylvatic transmission cycles, and for discovery of reservoir hosts. Thus, expression of *L. tarentolae* recombinant antigens representing epitopes specific to each of the *T. cruzi* lineages, may facilitate comprehensive epidemiological investigations. Our results encourage *L. tarentolae* expression to improve efficacy of candidate diagnostic antigens.

Proteins have been preferentially pursued for decades as highly sensitive and specific diagnostic antigens, and as vaccine candidates. Perhaps most importantly, as recognised in recent research on malaria^39^ and bacteria^44^ we have indicated here that glycosylation cannot be ignored in the search for improved diagnostics or efficacious vaccines.

## Supplementary information

Supplementary Information 1.

Supplementary Legends.
