# Supplementary material for: Glycosylation of Trypanosoma cruzi TcI antigen reveals recognition by chagasic sera
Source: Sci Rep. 2020 Oct 2;10:16395. doi: 10.1038/s41598-020-73390-9 (PMC7532467; doi:10.1038/s41598-020-73390-9)
Supplement: Supplementary file 1 — Supplementary Information 1. [file 41598_2020_73390_MOESM1_ESM.pdf]

Glycosylation of *Trypanosoma cruzi* TcI antigen reveals recognition by chagasic sera.

Niamh Murphy[1]*, Barrie Rooney[2,3]*, Tapan Bhattacharyya[1], Omar Triana-Chavez[4], Anja Krueger[5], Stuart M. Haslam[5], Victoria O'Rourke[1], Magdalena Pańczuk[1], Jemima Tsang[1], Jack Bickford-Smith[1], Robert H. Gilman[6], Kevin Tetteh[1], Chris Drakeley[1], C. Mark Smales[2], Michael A. Miles[1]

[1] Faculty of Infectious & Tropical Diseases, London School of Hygiene & Tropical Medicine, London, UK.

[2] Centre for Molecular Processing, School of Biosciences, University of Kent, Canterbury, Kent, UK

[3] TroZonX17, Kent, UK

[4] Instituto de Biología, Universidad de Antioquia, Medellín, Colombia

[5] Department of Life Sciences, Imperial College London, SW7 2AZ, London, UK

[6] Department of International Health, Johns Hopkins Bloomberg School of Public Health, Baltimore, USA.

* These authors contributed equally.

Fig S1

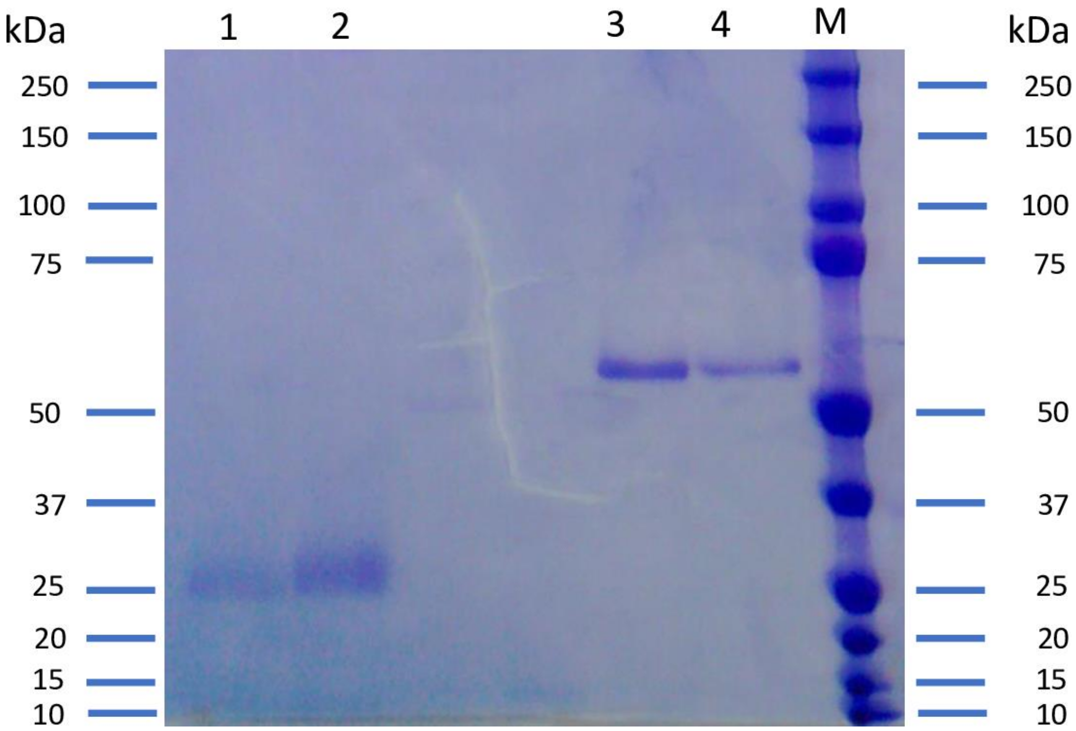
kDa
1
2
3
4
M
kDa
250
150
100
75
50
37
25
20
15
10
250
150
100
75
50
37
25
20
15
10

Fig S2

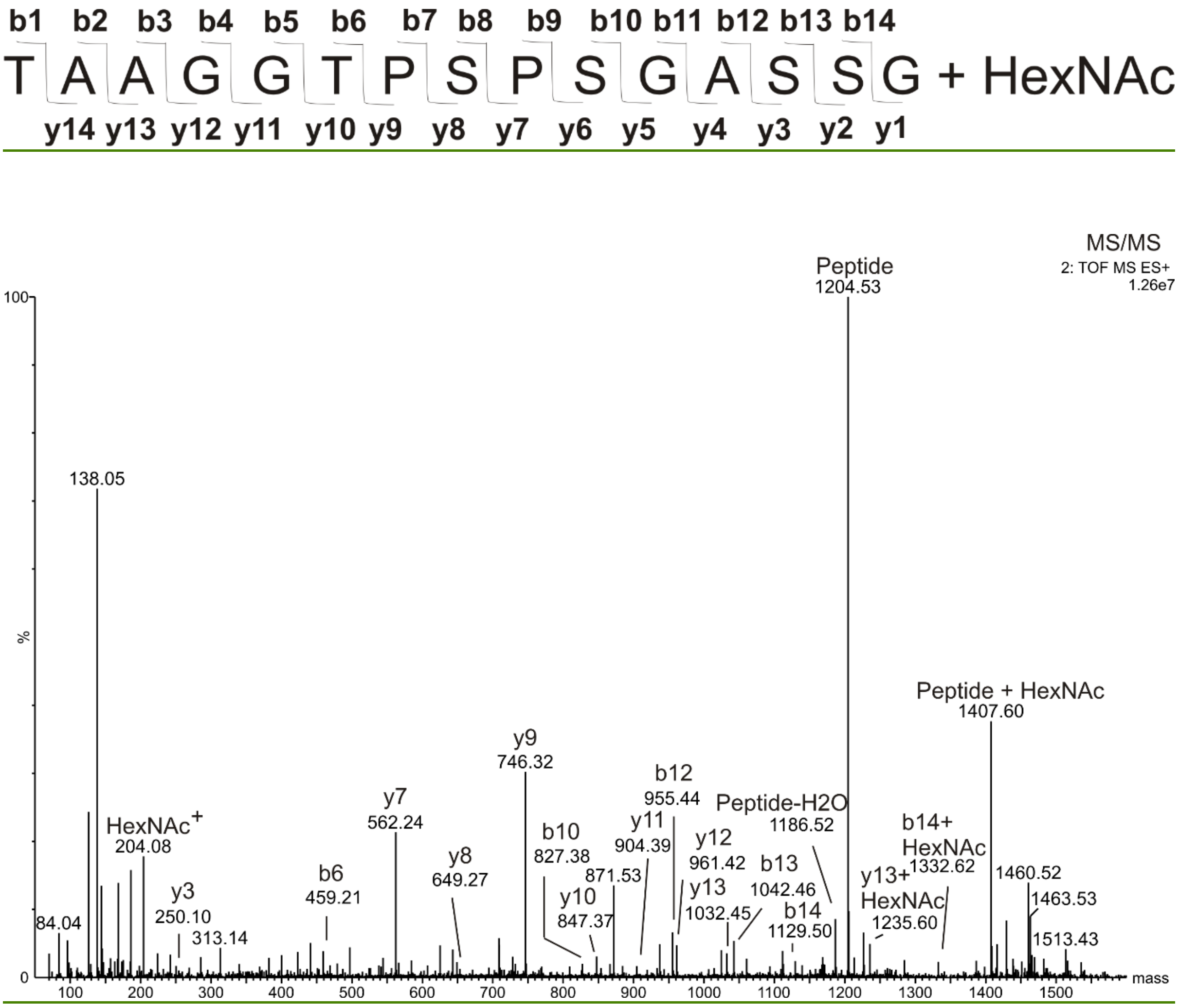
b1 b2 b3 b4 b5 b6 b7 b8 b9 b10 b11 b12 b13 b14
T A A G G T P S P S G A S S G + HexNAc
y14 y13 y12 y11 y10 y9 y8 y7 y6 y5 y4 y3 y2 y1
MS/MS
2: TOF MS ES+
1.26e7
Peptide
1204.53
138.05
%
Peptide + HexNAc
1407.60
y9
746.32
b12
955.44
Peptide-H2O
1186.52
y7
562.24
y11
904.39
b14+
HexNAc
1332.62
HexNAc+
204.08
b10
827.38
y12
961.42
b13
1042.46
y8
649.27
871.53
1460.52
b6
459.21
y13
1032.45
y13+
HexNAc
1235.60
y3
250.10
y10
847.37
b14
1129.50
1463.53
84.04
313.14
1513.43
100
0
mass
100 200 300 400 500 600 700 800 900 1000 1100 1200 1300 1400 1500
