# Supplementary material for: Glycosylation of Trypanosoma cruzi TcI antigen reveals recognition by chagasic sera
Source: Sci Rep. 2020 Oct 2;10:16395. doi: 10.1038/s41598-020-73390-9 (PMC7532467; doi:10.1038/s41598-020-73390-9)
Supplement: Supplementary file 2 — Supplementary Legends. [file 41598_2020_73390_MOESM2_ESM.docx]

**Supplementary Figure S1**. Full image of Coomassie blue stained gel of purified gTSSA-I.

**Supplementary Figure S2: Recombinant TSSA-I is glycosylated.** Glycoproteomics analysis of the TAAGGTPSPSGASSG glycopeptide derived from TSSA-I antigen. LC ES-MS (MS^e^ mode) data containing the [M+H]^+^ molecular ion found at m/z 1407.60 and interpreted as a TAAGGTPSPSGASSG glycopeptide substituted with a single HexNAc residue.
